# Drug Abuse Ontology to Harness Web-Based Data for Substance Use Epidemiology Research: Ontology Development Study

**DOI:** 10.2196/24938

**Published:** 2022-12-23

**Authors:** Usha Lokala, Francois Lamy, Raminta Daniulaityte, Manas Gaur, Amelie Gyrard, Krishnaprasad Thirunarayan, Ugur Kursuncu, Amit Sheth

**Affiliations:** 1 AI Institute, University of South Carolina Columbia, SC United States; 2 Department of Society and Health, Mahidol University Salaya Thailand; 3 College of Health Solutions, Arizona State Univeristy Phoenix, AZ United States; 4 Department of IoT and AI, Trialog Information Technology & Services Ile-de-France France; 5 Department of Computer Science & Engineering, Wright State University Dayton, OH United States

**Keywords:** ontology, knowledge graph, semantic web, illicit drugs, cryptomarket, social media

## Abstract

**Background:**

Web-based resources and social media platforms play an increasingly important role in health-related knowledge and experience sharing. There is a growing interest in the use of these novel data sources for epidemiological surveillance of substance use behaviors and trends.

**Objective:**

The key aims were to describe the development and application of the drug abuse ontology (DAO) as a framework for analyzing web-based and social media data to inform public health and substance use research in the following areas: determining user knowledge, attitudes, and behaviors related to nonmedical use of buprenorphine and illicitly manufactured opioids through the analysis of web forum data Prescription Drug Abuse Online Surveillance; analyzing patterns and trends of cannabis product use in the context of evolving cannabis legalization policies in the United States through analysis of Twitter and web forum data (eDrugTrends); assessing trends in the availability of novel synthetic opioids through the analysis of cryptomarket data (eDarkTrends); and analyzing COVID-19 pandemic trends in social media data related to 13 states in the United States as per Mental Health America reports.

**Methods:**

The domain and scope of the DAO were defined using competency questions from popular ontology methodology (101 ontology development). The 101 method includes determining the domain and scope of ontology, reusing existing knowledge, enumerating important terms in ontology, defining the classes, their properties and creating instances of the classes. The quality of the ontology was evaluated using a set of tools and best practices recognized by the semantic web community and the artificial intelligence community that engage in natural language processing.

**Results:**

The current version of the DAO comprises 315 classes, 31 relationships, and 814 instances among the classes. The ontology is flexible and can easily accommodate new concepts. The integration of the ontology with machine learning algorithms dramatically decreased the false alarm rate by adding external knowledge to the machine learning process. The ontology is recurrently updated to capture evolving concepts in different contexts and applied to analyze data related to social media and dark web marketplaces.

**Conclusions:**

The DAO provides a powerful framework and a useful resource that can be expanded and adapted to a wide range of substance use and mental health domains to help advance big data analytics of web-based data for substance use epidemiology research.

## Introduction

### Background

Illicit drug use is a complex social phenomenon generating a variety of public health issues that affect individuals and their communities. In its 2020 report, the United Nations Office on Drugs and Crime estimated that 5.4% of the world population used illicit drugs in 2018 while 0.7% of the whole population is affected by substance use disorder [1]. Individuals affected by substance use disorder are at risk of experiencing a variety of adverse psychiatric and physical health effects such as unintentional overdoses or disease infections (eg, HIV and hepatitis C). Individual drug use also potentially impacts the well-being of others, affecting local communities and neighborhoods [2], which in turn creates the contextual conditions and social determinants linked to individual drug use initiation [3]. Although cannabis remains by far the most consumed illicit drug with more potent forms potentially linked to adverse consequences [4], opioid and amphetamine-type drugs remain more frequently associated with psychiatric and physical harms [5].

Although illicit substance use represents an endemic phenomenon affecting modern societies, recent years have seen radical and rapid changes in terms of the variety of substances available, the growing role played by the internet, and the decriminalization or legalization of several illicit substances in an increasing number of countries. For example, the European Monitoring Centre for Drugs and Drug Addiction has identified and listed approximately 400 novel psychoactive substances since 2015 [6], while cryptomarkets located on the dark net have become increasingly important platforms for the distribution of novel psychoactive substances and other illicit or prescription drugs [7,8]. These changes call for more timely methods of data collection, allowing the monitoring of both demand and supply sides. In this ever-changing environment, user-generated content on illicit drug use shared on social media represents a rich source of unsolicited and unfiltered self-disclosures of attitudes and practices related to substance use [9]. Furthermore, web-based sources of distribution can be harnessed to provide updates on the illicit drug supply trade and new trends [10].

These unfiltered web-based communications and advertisements offer a rich source of data sensitive to changing and emerging drug use trends, and can be used to complement and enhance existing epidemiological surveillance systems.

Semantic web-based approaches play a key role in enhancing and improving big data analytics for such complex domains as substance use. The semantic web is an extension of the web in which a set of design principles and technologies have been created to capture the meaning of information [11]. An ontology is defined as a specification of shared concepts and relationships among them, consisting of a schema and a knowledge base of instances [12].

Ontologies also play key roles in the development of (1) semantic web applications, (2) semantic annotation of data, and (3) tools for querying and reasoning [13]. However, to apply semantic web tools effectively, there is a need for a domain-specific ontology to represent the main entities of value described in the social media posts and their relationships [14].

There has been a broad range of research developing ontologies for social media data. For instance, the work proposed by Kim et al [15] aimed to develop an ontology dedicated to obesity for investigating obesity-related social media posts and detecting sentiments, emotions, and opinions posted on specific social media. Their ontology was evaluated by mapping concepts from ontology with similar terms found in tweets related to obesity, and is only limited to 8 superclasses related to broader perspectives of any biomedical ontology. This study is limited to social media posts for improving upon the ontology, and the keywords are vastly distributed among the top 2 obesity types (abdomen and thigh) and top 3 management types (diet, exercise, and drug therapy) and are only limited to the general population in social media.

There are fewer ontologies related to the domain of mental health. For example, Jung et al. [16] proposed to design an ontology using an entity-attribute-value triplet data model dedicated to adolescent depression in order to analyze related social media. This ontology was developed using clinical guidelines and unstructured social media posts with 777 terms divided into *risk factors, signs and symptoms, screening, diagnosis, treatment, and prevention*. This work is mainly limited to the extraction of data solely from adolescent depression-related social media posts.

Several prior ontologies were developed for the analysis of the prescription drug domain. For example, the prescription drugs ontology [17] aims at improving the semantics of drug prescriptions and prospectively enabling the interoperability of prescription data by reusing classes and object properties from the information artifact ontology [18], the ontology for biomedical investigations [19], the ontology for general medical science [20], the ontology for medically related social entities [21], and the drug ontology [22]. However, these ontologies focus on medical uses of prescribed drugs and do not include concepts or slang terms related to the use of illicit drugs and addiction.

As the opioid crisis has deepened in recent years, efforts to analyze the opioid research on social media and make policy decisions have intensified. In a recent study, a specific knowledge graph called Opioid Drug Knowledge Graph (ODKG) [23] was developed to capture opioid-related drugs and related entities in eHealth records. As the drug abuse ontology (DAO) also contains information about opioid-related drugs, we compared the ODKG and DAO in terms of their coverage of relevant entities in opioid-related social media corpus (Twitter) and observed that the DAO outperformed the ODKG by order of magnitude. As the DAO was designed to also cover slang terms that are common in social media, it performed well by retrieving 7 million more tweets than the ODKG (2 million) from a resource of 1.2 billion crawled tweets during the COVID-19 pandemic [24].

The key aims of this paper were to describe the process of development, evaluation, and application of the DAO to facilitate and enhance social media and web-based analytics for substance use epidemiology research. This paper describes the process of DAO development in the context of 4 research projects out of which 3 are National Institutes of Health (NIH)–sponsored studies that aimed to harness web-based and social media data for substance use epidemiology research: (1) Prescription Drug Abuse Online Surveillance (PREDOSE) project that aimed to characterize user knowledge, attitudes, and behaviors related to nonmedical use of buprenorphine and other illicitly manufactured opioids through the analysis of web forum data [25-27]; (2) eDrugTrends project that focused on patterns and trends of cannabis product use in the context of evolving cannabis legalization policies in the United States through the analysis of Twitter and web forum data [28-32]; (3) eDarkTrends project that aimed to identify availability trends of novel synthetic opioids through the analysis of crypto market data [33-35]; and (4) COVID-19 pandemic trends in social media data related to 13 states in the United States and its mental health impact.

The terminology related to machine learning (ML), natural language processing (NLP), and ontology design used in this paper is organized alphabetically in Textbox 1.

**Descriptions of machine learning (ML), natural language processing (NLP), and ontology terms used in this paper.**

**Terminology and description**

- 101 ontology [36]: the 101 ontology is a guideline to create an ontology and offers step by step process. It leverages the authors’ experiences developing and maintaining ontologies in several ontology environments like Protégé.
- Bootstrap and bagged random Forest with contextual features (BRF-CF): Random Forest is one of the most popular ML algorithms. It is a type of ensemble ML algorithm called bootstrap or bagging.
- Class, data property, individual count: these terms are used as the signatures for the imports closure of the active ontology. In other words, the number of distinct classes, object properties, data properties, and individuals are mentioned in the ontology. The numbers here include built-in entities, such as owl: Thing if they are explicitly mentioned in the ontology.
- Community Ontology Repository [37]: this is the repository of ontologies hosted by Earth Science Information Partner’s members that would let users try out semantic technologies, understand their benefits, and explore possible applications that used semantic resources.
- Depression and drug abuse BERT: BERT is a bidirectional encoder representations from transformers and is a transformer-based ML technique for NLP. We fine-tune BERT models on corpora that are representative of depression and drug abuse.
- DBpedia [38]: DBpedia is a crowd-sourced community effort to extract structured content from the information created in various Wikipedia projects.
- Diagnostic and Statistical Manual for Mental Disorders (DSM)-5: It is the taxonomic and diagnostic manual developed and published by the American Psychiatric Association. It is an authoritative guide for mental health care professionals in the diagnosis of mental disorders.
- Entity, concept: the entity is referred to as an encompassing concept for classes, individuals, and properties. Concept and class are simply synonyms.
- F1 score: It is the weighted average of precision and recall. This score takes both false positives and false negatives into account. F1 is usually more useful than accuracy score.
- False positive, true positive: a false alarm is also known as a false positive. A false positive is a result that indicates a given condition exists when it does not. For example, the model indicates that cannabis can cause pain when it does not cause pain. A true positive is an outcome where the model correctly predicts the positive class. Similarly, a true negative is an outcome where the model correctly predicts the negative class. A false positive is an outcome where the model incorrectly predicts the positive class.
- Horizontal linguistic features, vertical linguistic features, fine-grained features: while training an ML model, we organized our feature set into 3 broad groups: horizontal linguistic features, vertical linguistic features, and fine-grained features. Contextual Features (or embedding of a social media post) with Modulations (CFwM) and without Modulations (CFw/oM) are 2 additional feature set created using Word2Vec.
- Ontology metrics [39]: the metrics list the numbers for structures and representation of ontology in Protégé as it is the most widely used tool to create an ontology. Axioms associate class and properties and are a combination of logical and nonlogical attributes. The number of distinct classes, object properties, data properties, and individuals reported is focused on the evaluation of the structure of DAO.
- Oops (ontology pitfall scanner), vapor, triple checker [40]: these are Semantic Web (SemWeb) validation or documentation tools that help to improve ontologies. Oops detect common pitfalls in ontology automatically and provide recommendations to fix them.
- Owl file: the W3C web Ontology Language is a SemWeb language designed to represent rich and complex knowledge about things, groups of things, and relations between things.
- PerfectO methodology [40]: PerfectO references, classifies, and provides tools to encourage SemWeb best practices to achieve semantic interoperability by focusing on ontology improvement.
- Precision, recall: precision is the proportion of times that when you predict it is positive and it actually turns out to be positive, whereas recall is like accuracy over just the positives—it is the proportion of times you labeled positive correctly over the number of times it was actually positive.
- Protégé: protégé is a free, open-source ontology editor and framework for building intelligent systems.
- SEDO [41]: It stands for Semantic Encoding and Decoding Optimization. It is a procedure to modulate the word embedding (vectors) of a word. SEDO modulates the embeddings of each word in the Reddit content of the user based on the proximity of the word to the Diagnostic and Statistical Manual for Mental Disorders-5th edition category.
- Vanilla BERT: Vanilla BERT is a variation of the attention-based BERT model and provides a pretrained starting point layer for neural networks.
- WebVOWL [42]: It is a web application for the interactive visualization of ontologies which is one of the ontology visual representations.

### Evolution of the DAO

As social media and other web resources play an increasingly important role in health-related knowledge and experience sharing [43], there is a need for an ontology explicitly dedicated to the domain of substance use research. The DAO was developed to formalize concepts, entities, and relationships relevant to the domains of addictions and mental health to harness its use on social media data. Our approach, built on the integration of semantic web technologies, enhances traditional ML and NLP techniques for automatic extraction and representation of relevant data and facilitates analysis and interpretation related to the specific goals of each study.

### Prescription Drug Abuse Online Surveillance

**Figure 1:**
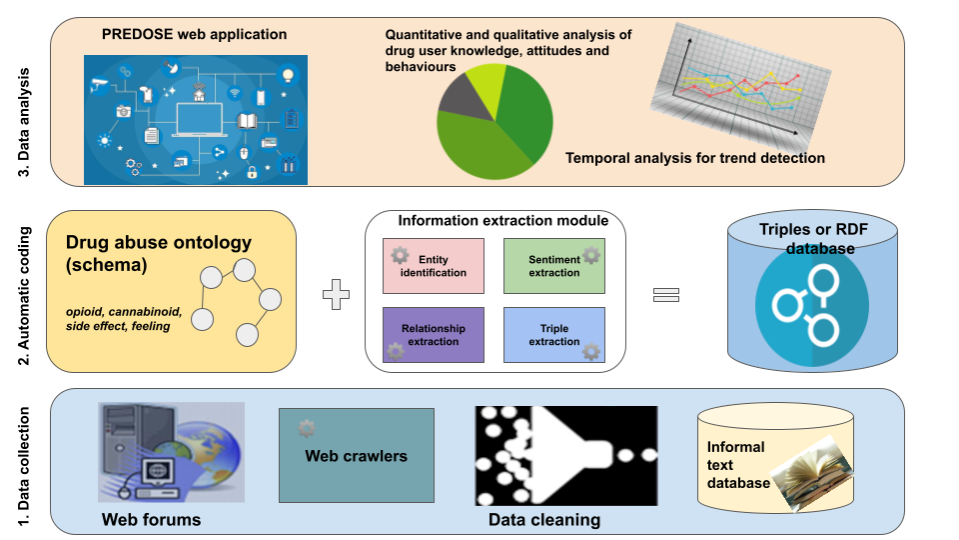
Use of the drug abuse ontology within Prescription Drug Abuse Online Surveillance (PREDOSE). RDF: Resource Description Framework.

This study focuses on web forum data related to the nonmedical use of buprenorphine [26,27] approved in late 2002 by the United States Food and Drug Administration for the treatment of opioid addiction. Use of buprenorphine was defined as nonprescribed when used without medical supervision. Although there is always a level of uncertainty in disambiguating prescribed versus nonprescribed use in web-based discussions, some of the questions and practices shared by individuals provided indicators about nonprescribed use (eg, saying that Suboxone was obtained from a friend; that *bupe* was snorted; or that it was cut up and used in smaller amounts). Buprenorphine (Suboxone, Subutex, etc) is the only controlled substance that may be prescribed for the treatment of opioid addiction by a licensed physician in an office-based setting. The overall purpose of PREDOSE was to study user-generated web forum discussions about the illicit use of Suboxone (buprenorphine or naloxone), Subutex (buprenorphine), and other buprenorphine products by applying novel information processing techniques to facilitate qualitative and quantitative analysis [26]. Along with Twitter and Reddit, we also used 3 web forums that provided venues for people to freely share drug use experiences and post questions, comments, and opinions about different drugs. One of these web forums used in our research was Bluelight [44] (please note that in compliance with Institutional Review Board guidelines at Wright State University, the names of the other 2 forums have not been disclosed in this paper). Our team has developed a research collaboration with the Bluelight team and was able to obtain deidentified data updates directly from Bluelight. Data from these forums were collected using custom-built web crawlers. We chose to study buprenorphine because there was at that time (2011-2012) a growing body of evidence that buprenorphine was used and that there was relatively little knowledge about the patterns and trends of its nonmedical use in the United States. As buprenorphine use is linked to a broader domain of illicit opioid use and addiction, the initial versions of the DAO included detailed representation of the opioid class drugs, including slang and brand name terminology. The DAO developed for the PREDOSE project also included other classes of drugs, such as cannabis and stimulant-type drugs, because polysubstance use is common among illicit opioid users. Figure 1 [26] demonstrates the use of the DAO ontology within our PREDOSE architecture, which comprises three main modules:

1. Data collection module that collected approximately 1 million posts (1,066,502) from 35,974 users.
2. Automatic coding module that semantically annotated the posts using the DAO ontology.
3. Data analysis and interpretation module to visualize the keywords (eg, loperamide and buprenorphine) found within posts and referenced within the DAO ontology.

### eDrugTrends

This is our second project that received funding from NIH and National Institute on Drug Abuse (NIDA) in 2014 [45]. This study focused on social media data related to cannabis and synthetic cannabinoid use in the context of evolving cannabis legalization policies in the United States. The aim of this study was to develop eDrugTrends, a comprehensive software platform for semiautomated processing and visualization of thematic, sentiment, spatiotemporal, and social network dimensions of social media data (Twitter and web forums) on cannabis and synthetic cannabinoid use. The study also aimed to (1) identify and compare trends in knowledge, attitudes, and behaviors related to cannabis and synthetic cannabinoid use across United States regions with different cannabis legalization policies using Twitter and web forum data and (2) analyze social network characteristics and identify key influencers in cannabis and synthetic cannabinoid–related discussions on Twitter. For addressing these aims of the eDrugTrends platform, the DAO was expanded further to include a more comprehensive representation of emerging cannabis products, synthetic cannabinoid products, health-related consequences, and mental health conditions.

### eDarkTrends

This is the third project using the DAO. This study was funded through the NIH and NIDA time-sensitive mechanism [46], which started in 2017. The eDarkTrends project was orientated toward novel synthetic opioids, such as illicitly manufactured fentanyl that have emerged over the past few years and were and still are significant contributors to the increase in unintentional opioid-related overdose mortality in the United States [35,47,48]. However, epidemiological surveillance on cryptomarket data was limited at the time (2017). The study’s overall goal was to harness cryptomarket data to conduct surveillance of illicit fentanyl, fentanyl analog, and other novel synthetic opioid availability trends over time and identify new substances as they emerge in the Darknet environment. Ultimately, eDarkTrends aimed at providing a powerful tool for epidemiological surveillance, enhancing the capacities of early warning systems to capture changes in the fentanyl and other illicit synthetic opioid supply and availability. For addressing the specific needs of the project, the DAO was further expanded to include a comprehensive and detailed representation of novel illicit synthetic opioid domains (eg, carfentanil, furanyl fentanyl, U-47700, and MT-45).

### COVID-19 Pandemic

In addition, we applied the DAO on COVID-19 social media data analysis to analyze the social media data related to the pandemic. The intent is that the COVID-19 pandemic has alleviated community-wide depression and has led to increased drug use [49]. The impact of the COVID-19 pandemic on mental health was investigated in recent studies [50-52]. For this, we proposed a novel framework for assessing the spatiotemporal-thematic progression of depression, drug use, and informativeness of the underlying news content across different states in the United States [53]. The DAO is used along with the Medical Subject Headings terms hierarchy in the Unified Medical Language System, the Diagnostic and Statistical Manual for Mental Disorders-5th edition (DSM-5) lexicon [41], which are collectively referred to as the Mental Health and Drug Abuse Knowledge base (MHDA-Kb) to spot additional entities.

## Methods

### Overview

The ontology was manually developed by the domain expert coauthors (FL and RD), who used a range of sources, including (1) key epidemiological data sources and reports accessible through the NIDA [54], Drug Enforcement Agency [55], European Monitoring Centre for Drugs Addiction [56], and RxNorm [57]; (2) prior peer-reviewed publications related to illicitly manufactured opioids, cannabis, and other drugs [58-61]; and (3) ongoing manual assessment and examination of web-based social media sources related to selected substances [25,27,62]. Sources of types 1 and 2 provided primary concepts while sources of type 3 were important in identifying alternative concepts, including synonyms and street names. To develop the DAO, we followed the well-known 101 ontology development methodology [63]. The 101 method includes (1) determining the domain and scope of ontology, (2) reusing existing knowledge, (3) enumerating important terms in ontology, and (4) defining the classes and their properties and creating instances of the classes.

### Design

**Figure 2:**
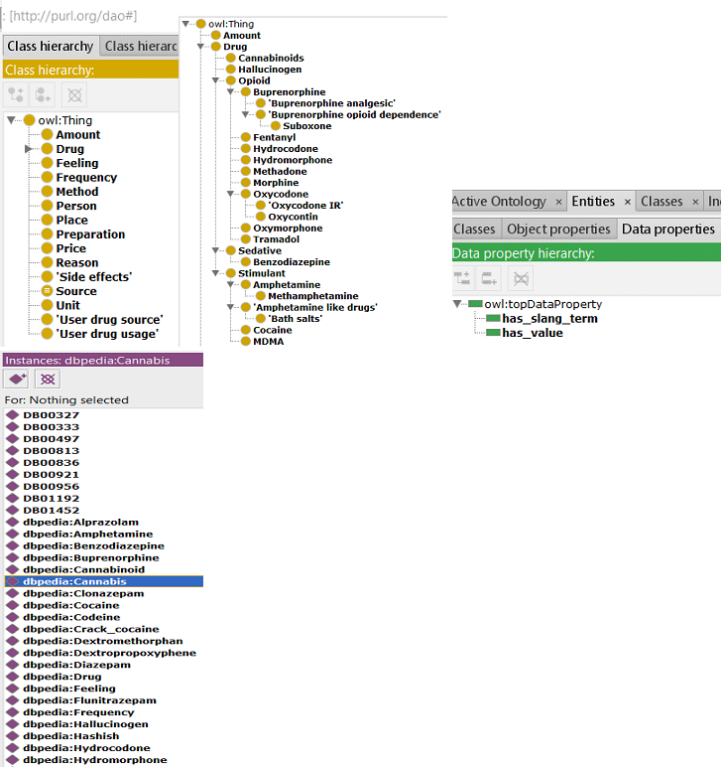
Drug abuse ontology in Protégé (concepts, object properties, data properties, and instances).

Figure 2 provides an overview of the DAO ontology. Protégé [64], a popular ontology editor, was used to build the ontology as a tree of subclasses. The ontology was designed as a catalog of concepts related to substance use. Hence, classes of psychoactive substances (eg, cannabinoids and opioids) were created and populated with subtypes of substances (eg, morphine and fentanyl). Each substance was defined by its name and, when applicable, information regarding its pharmaceutical or brand name (*has_brand_name*), slang or street name (*has_street_name*), and chemical designation (*has_chemical_formula*) were added. This latter information was collected through different sources: pharmaceutical or brand names were based on existing medical or pharmacological dictionaries, slang or street names were based on the domain knowledge of the second and third authors (RD and FL), and chemical designations mostly concerned synthetic cannabinoid receptor agonists and were based on academic literature as well as on seizure data (eg, the National Forensic Laboratory Information System and Europol). The DAO was also enhanced with concepts and slang terms related to those concepts regarding unit (eg, *caps, ml, and bottle),* purity, and form of preparation (*eg, crush and eyeball)* to enable the identification and analysis of triple in text content [65]. For example, one instance of the drug *Morphine* is *Poppy_Tea*, which has the slang terms *Pod* and *Poppy_Pods* used on social media.

### Instantiation

**Figure 3:**
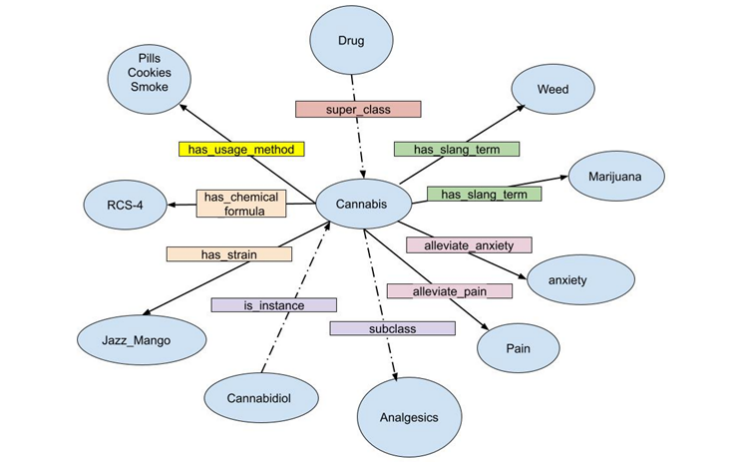
Web-based visualization of OWL ontologies (WebVOWL) representation of the drug abuse ontology, focused on the cannabis concept. RCS-4: 1-pentyl-3-(4-methoxybenzoyl)indole.

This is defined as creating instances of classes in a hierarchy. The instance of a class has its own class and fills a value. The instance has its own properties. For example, *Fentanyl* belongs to the class *Opioid* and has its own properties such as *has_brand_name, has_synonym, has_slang_term,* etc. The DAO ontology reuses instances from the DBpedia data set [66] (eg, buprenorphine). Figure 3 is the WebVOWL (web application for the interactive visualization of ontologies) representation of the DAO focused on the entity Cannabis derived from the visual data web [67]. Figure 2 shows the tree of drug names implemented as a web ontology format (owl) file within the DAO ontology. In Figure 2, entities, object properties, instances, and data properties are represented in yellow, green, and purple tags, respectively, which clearly depict the nature of classes, instances, hierarchies, and relationships for each entity.

### Ethics Approval

This research is done in compliance with institutional review board guidelines at Wright State University. The names of the selected websites have not been disclosed in this manuscript. Our project involves analysis of Twitter data that is publicly available and that has been anonymized. It does not involve any direct interaction with any individuals or their personally identifiable data. Furthermore, our data set does not include any interaction with human participants. Our data set does not contain any images as per our data use safety agreement. Thus, this study was reviewed by the Wright State University Institutional Review Board and received an exemption determination.

## Results

### Evaluation

The DAO ontology was evaluated following the semantic web best practices recognized by the International Semantic Web Conference Resource Track guidelines [68], which provide the following criteria: (1) impact, (2) reusability, (3) design and technical quality, and (4) availability. We have also followed the PerfectO methodology [40], which synthesizes a set of additional best practices and eases their achievements [69]. We have discussed the results of applying the following criteria to our DAO:

1. Impact and reusability: the DAO has been exploited in 4 scenarios, as mentioned earlier. Automatic documentation can be provided using the Live OWL documentation environment [70], and the DAO documentation is available in Community ontology repository [71].
2. Design, technical quality, and availability: the design of the ontology is available on the web as a graph visualization using web-based visualization of ontologies (WebVOWL) [72,73]. We improved the ontology using Oops (Ontology Pitfall Scanner) tools that automatically detect common pitfalls and provide recommendations to fix them. Oops loaded with the DAO can be tested on the web [71,74]. The Linked data validator, Vapour tool integrated with the DAO [75] was used to check dereferencing uniform resource identifier and content negotiation. Finally, Resource description framework Triple-Checker checks whether the existing ontologies have been correctly used within our DAO [76].
3. Ontology metrics: the DAO was also evaluated, as shown in Table 1, with respect to several ontology metrics [77]. The metrics list the numbers for the structures and representation of ontology in Protégé, as it is the most widely used tool to create ontology [78]. Axioms associate class and properties and are a combination of logical and nonlogical axioms [79]. The number of distinct classes, object properties, data properties, and individuals reported in Table 1 are focused on the evaluation of the structure of the DAO.

**Table 1:** Drug abuse ontology metrics: the ontology metrics view displays entity and axiom count for the axioms in the active ontology [39].

Metric		Count, n	Description
**Ontology metrics**			
	Axioms	4876	Combined logical and nonlogical axiom count
	Logical axiom count	3478	The number of logical axioms
	Declaration axioms count	1185	The number of declaration axioms
	Class count	316	The number of distinct classes, object properties, data properties and individuals that are mentioned in the ontology
	Object property count	12	The number of distinct classes, object properties, data properties and individuals that are mentioned in the ontology
	Data property count	13	The number of distinct classes, object properties, data properties and individuals that are mentioned in the ontology
	Individual count	845	The number of distinct classes, object properties, data properties and individuals that are mentioned in the ontology
**Class axiom**			
	SubClassOf	313	The number of SubClassOf axioms in the ontology. A subclass axiom states that a class is a subclass of another class
**Individual axioms**			
	Data property assertion	2317	A data property assertion states that the individual is connected by the data property expression to the literal.
	ClassAssertion	830	A class assertion states that the individual is an instance of the class expression.
	AnnotationAssertion	213	An annotation assertion states that the annotation subject is an anonymous individual with the annotation property and value.

The subsequent sections demonstrate the results with the DAO in different platforms and the evolution of the DAO with each use case.

### The DAO Within PREDOSE

**Figure 4:**
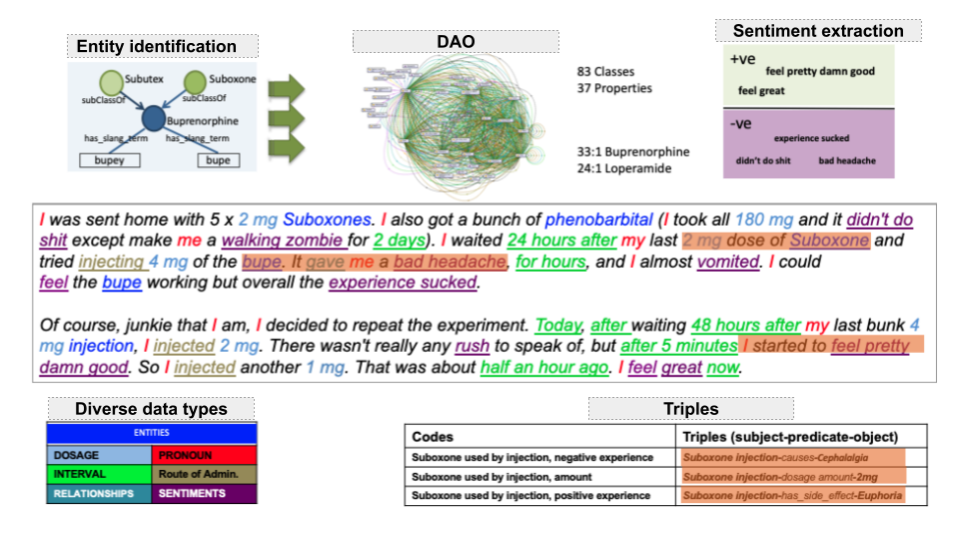
Automatic annotation of texts with the drug abuse ontology (DAO) [80].

**Figure 5:**
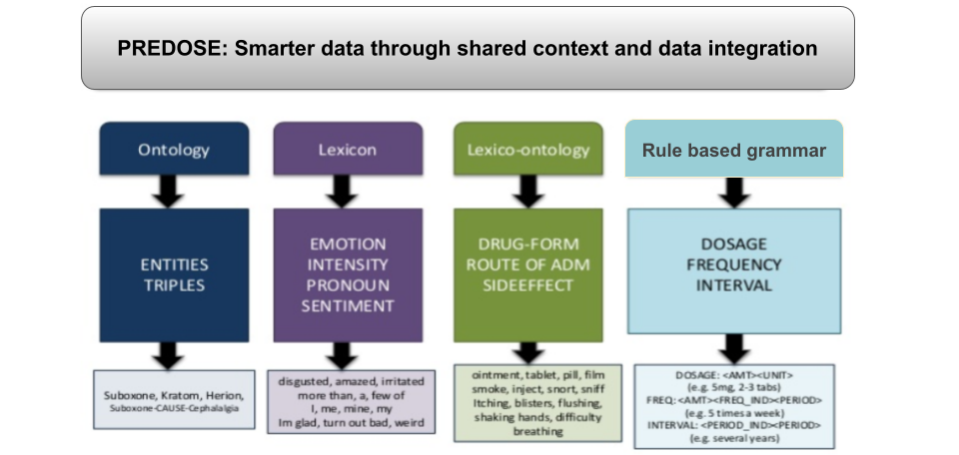
Benefits of ontologies with lexicons and rule-based grammar [80].

**Figure 6:**
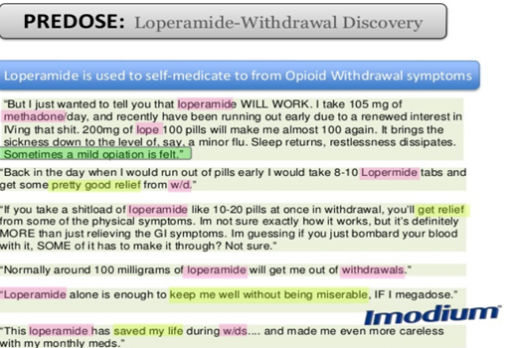
Loperamide discovery and its use in self-medication for opioid withdrawal.

Figure 4 [26,80] describes how the texts are automatically annotated using the DAO. In the text shown in Figure 4, we identify drug entities, dosage, time interval, route of administering the drug, etc. In the DAO, buprenorphine is defined as the subclass of *Subutex* and *Suboxone*. It has the slang terms *Bupe* and *Bupey*. The term *Bupe* identified in the text would not have been possible without defining it as a slang term in the DAO. The DAO is capable of mapping units (eg, mg→MILLIGRAM) and slang terms (eg, bupe— buprenorphine) based on a lexical lookup in the ontology. Similarly, other concepts, such as the route of administration *injected*, are also identified in the text. In NLP-related tasks, such as lexical, semantic, and syntactic analysis of textual data, adding ontology works as an external source of knowledge in identifying triples and entities in data. Conceptualizing the domain in data acts as a prior requirement for processing further information (lexicon and rule-based grammar) about it [81] (Figure 5 [80]). When evaluating 601 web forum posts with the DAO, we achieved 84.9% precision and 72.5% recall in information extraction tasks. In particular, out of 3639 annotations, 2640 were predicted correct (true positives), whereas 683 slang terms are incorrect (false positives). As far as the recall is concerned, only 999 out of 3639 annotations are missed (false negatives) [26]. For triple extraction with the DAO, we achieved 33% precision across 197 evaluated triple patterns (66 were correct and 131 were incorrect). For relation extraction with the DAO, we achieved 36% precision across 183 phrases (66 were correct and 117 were incorrect). Another finding (Figure 6 [25]) is that our analysis of web forums with the DAO revealed that loperamide was widely used as a treatment for withdrawal symptoms related to opioid addiction, where buprenorphine and methadone are commonly prescribed. A total of 3 toxicology studies following this work led to a Food and Drug Administration warning in 2016 [25,82]. A video demo [83] on the PREDOSE platform is available on the web. The PREDOSE platform indicates a need for additional enhancements in information extraction and automated data coding techniques.

### eDrugTrends (Monitoring Drug Trends on Social Media)

**Figure 7:**
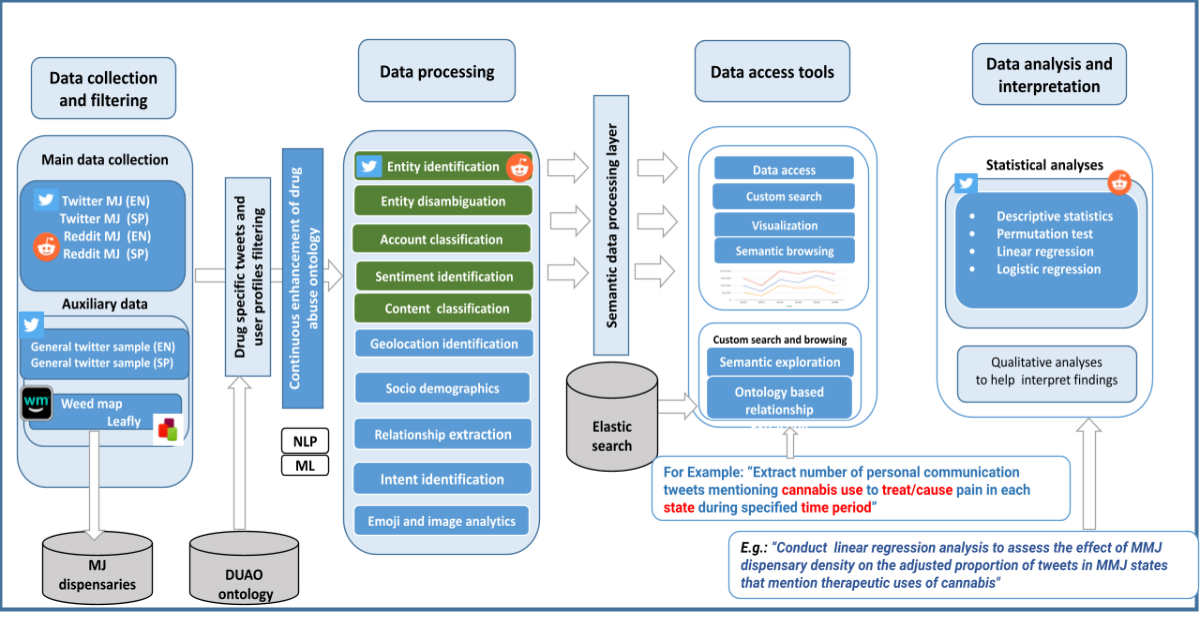
Architecture of the eDrugTrends project.

The eDrugTrends project aimed to analyze trends in knowledge, attitudes, and behaviors related to the use of cannabis and synthetic cannabinoids on web forums and Twitter [26,28-31]. Figure 7 [79] shows the application of the DAO ontology within the eDrugTrends architecture, which includes 4 stages: (1) data collection, (2) data processing, (3) data access tools for exploration and visualization, and (4) quantitative and qualitative analyses and interpretation. From the social science or substance use epidemiology perspective, the data processing and information extraction stages correspond with the coding task that prepares raw data for further analysis and interpretation. During data processing, the DAO came into the picture by playing an important role in identifying entities in the data that are exact names or synonyms or slang terms or street names of a drug. We generated embedding vectors using the DAO for domain-specific word embedding models and built an ML model to classify users by their types (individual, agency, and retailer) on Twitter by classifying their marijuana-related conversations [28]. We achieved this using multimodal embeddings extracted from people, content, and network views, achieving an 8% improvement over the empirical baseline [28]. We evaluated our approach using the average F1-score for each user type individual (P), informed agency (I), and retailer (R). The F1 scores for the individual classes P, I, and R were 95%, 42%, and 73%, respectively. The descriptive statistics of the training set at the Twitter user account level used for this study, which involved semantic filtering [84] using the DAO, are shown in Table 2.

**Table 2:** Descriptive information of user accounts on Twitter extracted using the drug abuse ontology [28].

Features	Personal accounts	Retail accounts	Informed agency	Total
Number of tweets	9836	1928	338	12,102
Number of profile pictures	4394	476	111	4981
Number of users with description	3884	461	108	4453
Number of retweets	955	24	964	1943
Number of mentions	94	6	307	407

### Enhancing the DAO With DSM-5

The motive for enhancing the DAO with DSM-5 is to provide actionable information to clinicians about the mental health of a patient in diagnostic terms for web-based interventions. We chose Reddit data for this study as the concepts, instances, and relations associated with drugs are semantically connected to mental health communications on social media, especially on Reddit. In our Reddit corpus, the drug use–related categories form a substantial portion (48%; corpus size is 2.5 million posts from 15 mental health subreddits by 268,104 users) of the data set in size. However, the DAO still lacked concepts directly related to mental health diagnostic disorders as defined in DSM-5 that are present in the International Classification of Diseases 10th edition [85], Systematized Nomenclature of Medicine-Clinical Terms [86], and DataMed [87]. In a recent study [41] on matching mental conditions of user posts on Reddit to DSM-5 diagnostic disorders, we enhanced the DAO with knowledge derived from DSM-5, which includes 20 chapters (Table 3), consistent with International Classification of Diseases 10th edition and NIH’s research domain criteria [88] for mental health. The enhanced DAO includes representations of mental health disorders and related symptoms that were developed following the DSM-5 classification [89]. For example, references for *Cannabis Use Disorder* include terms such as *addicted to cannabis*, *addicted to Marijuana*, and *Jazz_mango addict.* References to the feeling of *anxiety* or *anxious* include such terms as *antsy, worried,* and *agitated.* These lay terms were added to the DAO manually using synonym dictionaries and by manually examining Reddit conversations related to depression, anxiety, and other mental health conditions.

**Table 3:** Demonstration of improvement in the number of DSM-5^a^ category–related concepts being captured before and after including the DAO^b^ [41].

DSM-5 category	DSM-5–related concepts captured without the DAO, n	DSM-5–related concepts captured with the DAO, n
Dissociative disorder	20	20
Anxiety disorder	40	87
Substance use and addictive disorder	39	123
Schizophrenia spectrum	77	77
Sleep-wake disorder	14	19
Paraphilic disorders	14	14
Gender dysphoria	15	15
Neurodevelopmental disorders	25	53
Sexual dysfunctions	23	23
Personality disorders	76	98
Trauma and stressor related disorder	25	28
Disruptive, impulse, control, and conduct disorder	34	34
Psychotic disorders	85	87
Bipolar and related disorders	75	84
Elimination disorders	18	18
Depressive disorders	71	107
Obsessive-compulsive related disorder	43	60
Feeding and eating disorders	32	39
Neurocognitive disorders	80	80
Suicidal behavior or ideation	34	47

^a^DSM-5: Diagnostic and Statistical Manual for Mental Disorders-5th edition.

^b^DAO: drug abuse ontology.

**Figure 8:**
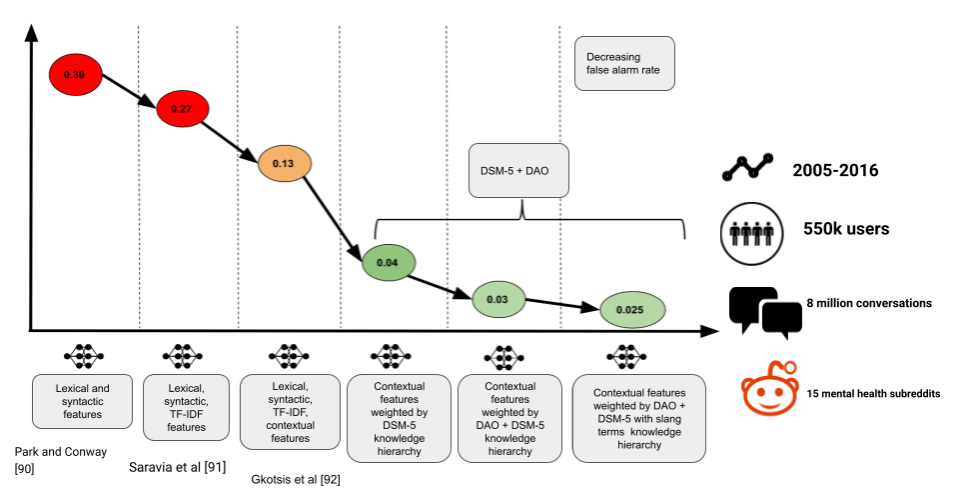
Results illustrating that domain-specific knowledge bases lower false alarm rates in identifying Diagnostic and Statistical Manual for Mental Disorders-5th edition (DSM-5) categories to tag posts in mental health subreddits. DAO: drug abuse ontology.

The DAO, curated and enhanced by DSM-5 concepts, was used in a weakly supervised setting to label Reddit posts with DSM-5 categories. In a comparative analysis with the state-of-the-art research by Park and Conway [90], Saravia et al [91], and Gkotsis et al [92], we observed that expansion of the DAO with DSM-5 helped improve the accuracy of our entity identification tools (reduced false positives by 92%). These results are shown in Figure 8. We further assessed the meaningfulness of the prediction through a reliability assessment with a domain expert, which gave an agreement score of 84%. In addition, the incorporation of slang terms from the DAO to match and process the informal social media data improved both coverage and recall (Table 4). Thus, we demonstrated that semantic weighting of contextual features from the content using the DAO and DSM-5 knowledge could significantly improve the robustness of the artificial intelligence system. As web-based content is mapped to a clinically acceptable vocabulary, the system brings in explainability. Furthermore, Table 3 shows the improvement in the number of concepts extracted from the DAO being captured in our Reddit Corpus that relate to DSM-5, 20 chapters before and after adding slang terms.

**Table 4:** Ablation study on contextual features and their modulation using SEDO^a^ weights generated from either DSM-5^b^ or its enrichment using the DAO^c^ and slang terms^d^.

Method (with horizontal linguistic features, vertical linguistic features, and fine-grained features)	Precision	Recall	F1-score
BRF^e^ with CF^f^	0.60	0.54	0.57
BRF-CF (SEDO weights generated from DSM-5 lexicon without the DAO)	0.87	0.77	0.82
BRF-CF (SEDO weights generated from DSM-5 lexicon with the DAO without slang terms)	0.87	0.80	0.83
BRF-CF (SEDO weights generated from DSM-5 lexicon without the DAO with slang terms)	0.85	0.82	0.83
BRF-CF (SEDO weights generated from DSM-5 lexicon with the DAO with slang terms)	0.88	0.83	0.85

^a^SEDO: Semantic Encoding and Decoding Optimization.

^b^DSM-5: Diagnostic and Statistical Manual for Mental Disorders-5th edition.

^c^DAO: drug abuse ontology.

^d^This table demonstrates the improvement of models with the enhanced DAO.

^e^BRF: balanced random forest.

^f^CF: contextual features.

The base model for the ablation study is a balanced random forest with horizontal linguistic features (number of definite articles, words per post, first-person pronouns, pronouns, and subordinate conjunctions), vertical linguistic features (number of part-of-speech tags, similarity between the posts, intrasubreddit similarity, and intersubreddit similarity), and fine-grained features (sentiment, emotion, and readability scores).

### eDarkTrends (Monitoring Drug Trends on Cryptomarkets)

**Figure 9:**
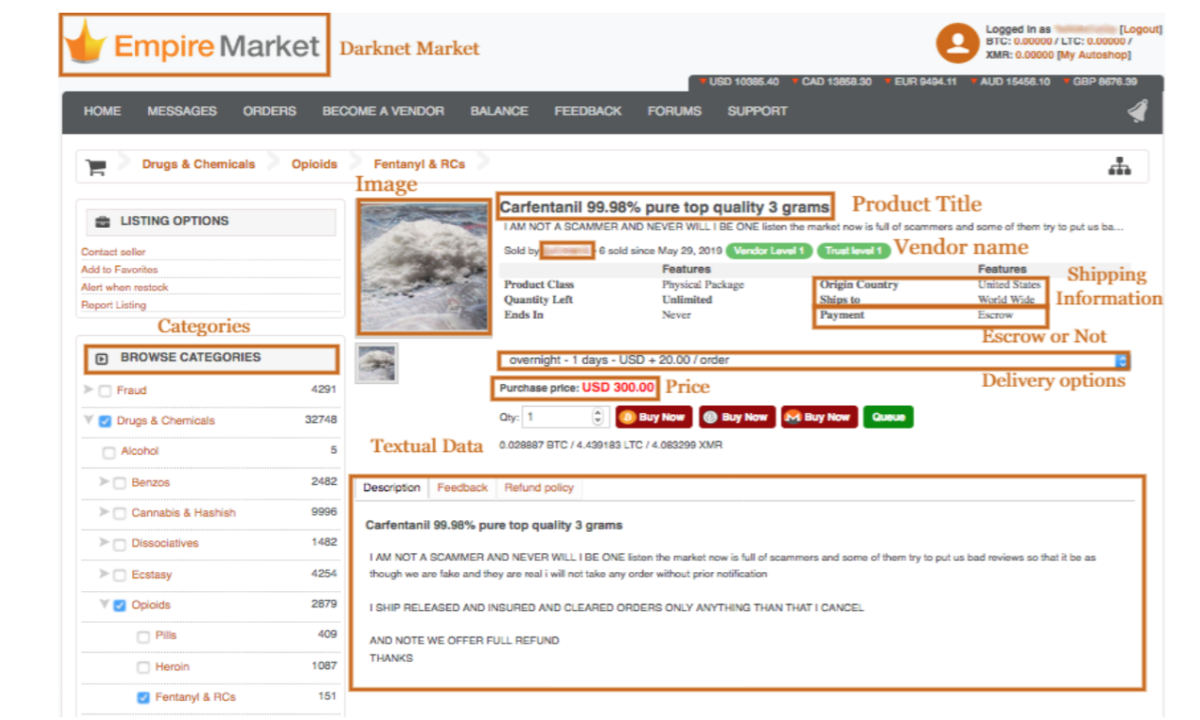
Screenshot of the Darknet marketplace.

The DAO also plays an essential role in identifying relevant entities and analyzing data from the Darknet cryptomarkets (eg, Agora, Dream Market, and Empire Market) to quantify and assess the availability of fentanyl, fentanyl analogs, and other novel synthetic opioids on the cryptomarkets [25,26]. The snapshot of the Darknet Marketplace is shown in Figure 9 [33]. The terms and slang terms associated with instances populating the DAO opioid subclass, as well as the dosage (eg, gram, mL, and ounce) and form (eg, tablet and powder) classes were compiled as regular expressions and used as expression patterns in the dedicated named entity recognition (NER) algorithm specifically designed for Darknet data [35]. The DAO was inductively augmented with abbreviations and terms specific to the cryptomarket environment (eg, fuff for fluoro-furanyl fentanyl or FE for finalize early) to ensure that only relevant data on novel synthetic opioids were collected. The NER allows capturing the types and quantities of novel synthetic opioids advertised on crypto markets; for example, the NER would provide the following information about the advertisement *FENTANYL TRANSDERMAL PATCHES 100 mcg per h* as class: fentanyl-type; name: fentanyl; dosage: 0.0001 g per h; form: transdermal. The results regarding the average numbers of fentanyl, fentanyl analogs, and other nonpharmaceutical synthetic opioids advertised on cryptomarkets identified are shown in Table 5. The crawls considered to obtain these results were the dark web posts collected from the Agora and Dream markets in the years 2015 and 2018 [35]. We also classified vendors on Darknet markets (Dream, Tochka, and Wall Street are the marketplaces used for this study) using the DAO. The summary of our findings related to unique vendors, substance, location, vendor descriptions, and the number of withdrawal transactions is shown in Table 6.

**Table 5:** Average number of fentanyl, fentanyl analogs, and other nonpharmaceutical synthetic opioids advertised on cryptomarkets extracted with the drug abuse ontology [34].

Types of substances			Average number of advertisements per day, by month (number of crawls)									
			Agora							Dream Market		
			March 2015		April 2015		May 2015		March 2018			April 2018
Fentanyl^a^			130		174		139		207			216
**Fentanyl analogs**												
	Acetyl fentanyl	44		39		41		3			1	
	Butyr fentanyl	12		10		17		6			7	
	Carfentanil	0		0		0		12			5	
	Furanyl fentanyl	0		0		1		31			39	
	Methoxy Acetyl fentanyl	0		0		0		14			14	
	4-fluroIsoButyr fentanyl	0		0		0		19			16	
	3-methoxyMethyl fentanyl	0		0		0		2			2	
	Total, fentanyl analogs	56		49		59		87			84	
**Other NP^b^ synthetic opioids**												
	U-47,700	5		4		5		0			3	
	W-18	5		4		5		0			0	
	MT-45	9		8		9		0			0	
	AH-7921	0		0		1		0			0	
	U-48,800	0		0		0		1			7	
	U-49,900	0		0		0		0			1	
	U-4TDP	0		0		0		0			4	
	U-50,488	0		0		0		8			4	
	MPF-47700	0		0		0		0			5	
	Total, other NP synth opioids	19		16		20		9			24	
Other opioids^c^			827		1061		1152		3211			3137
Total (any opioids)			1033		1300		1370		3512			3460

^a^Includes mentions of fentanyl, China white heroine, synthetic heroine, and mentions of pharmaceutical fentanyl such as Duragesic, fentanyl patches, and fentanyl transdermal system.

^b^NP: nonpharmaceutical.

^c^Includes mentions of heroin, opium, morphine and other types of pharmaceutical opioids (eg, hydrocodone, oxycodone, and hydromorphone) excluding pharmaceutical fentanyl.

**Table 6:** Summary of data set extracted from Darknet markets using the drug abuse ontology [33].

Marketplace	Withdrawal number of transactions	Bitcoin	US $ equivalent	Unique number of vendors	Unique number of substances	Unique number of locations	Unique number of descriptions
Dream	261	99.1503695	197,589.12	1448	852	356	16,800
Tochka	2990	0.70483642	5072.33	408	313	44	1829
Wall Street	7755	2.572515	18,729.40	466	290	29	1723

### COVID-19 Scenario

**Figure 10:**
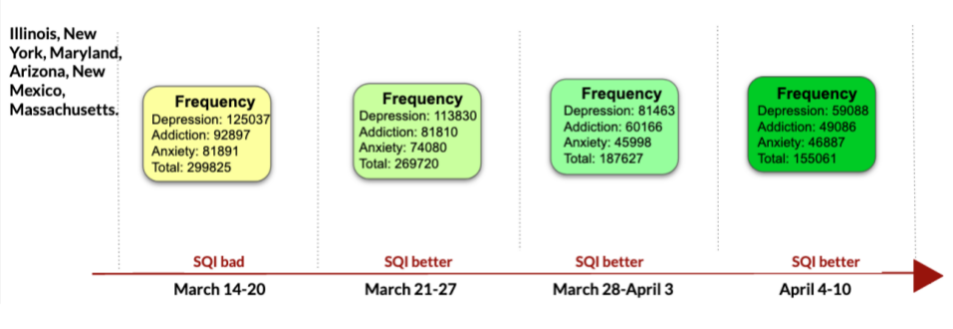
Social quality index (SQI) pattern of improvement in conditions as the decline in the number of tweets on depression, addiction, and anxiety.

We performed a spatiotemporal analysis of the psychological impact of the novel COVID-19 using approximately 1.2 billion tweets from January 1 to April 10, 2020 [93,94]. The concepts related to addiction and mental health in the COVID-19–related data were semiautomatically recognized using the entities and slang terms mentioned in the DAO. Approximately 90 related concepts and 140 slang terms were used to extract tweets mentioning illicit drug use, alcoholism, and pharmacological drug misuse. Furthermore, suicide risk factors such as insomnia and depression were observed in the tweets extracted using the DAO. Similarly, we studied the negative media exposure from approximately 700,000 news articles published during the COVID-19 pandemic by fine-tuning the bidirectional encoder representations from transformers (BERT) model with the DAO [53]. The 3 months (January, February, and March) in the year 2020 were considered for our earlier study, as this period had a huge COVID-19 spread as per the Mental Health America report [95]. We used 10 of the 13 states recognized as high-spread areas in this report. The 3 states that are not included in Table 7 are Washington, Wyoming, and Idaho. These 3 states were not included, as the related data were not present in our data set cohort. In this work, we reported the state-wise labels (ie, depressive, drug abusive, and informative) for each month using deep learning models vanilla BERT, depression BERT, and drug use BERT, as shown in Table 7. The definitions of these deep learning models are described in Textbox 1. This study is followed by analyzing the Social Quality Index, which aggregates mental health components (depression and anxiety), addiction, and substance use disorders, considering tweets in the period March to April 2020. The Social Quality Index and tweets for states Illinois, New York, Maryland, Arizona, New Mexico, and Massachusetts are shown in Figure 10 [94].

**Table 7:** Evaluation of BERT^a^ models for Mental Health America states over 3 months (January, February, and March 2020) [53,94].

Mental Health America states with depression and drug use	vanillaBERT (2020; months)	Druguse-BERT (2020; months)	Depression BERT (2020; months)
Tennessee	February and March	February and March	February and March
Alabama	February	February and March	February
Oklahoma	March	February and March	February and March
Kansas	February	January and February	January and February
Montana	March	February	February and March
South Carolina	March	March	February and March
Alaska	February and March	January, February, and March	February and March
Utah	March	March	March
Oregon	None	February	None
Nevada	February	February	February

^a^BERT: bidirectional encoder representations from transformers.

## Discussion

### Strengths and Limitations

The DAO is an ongoing project that can be continuously improved and expanded to handle additional topic areas and emerging substance use issues and trends. DAO development requires intensive, hands-on involvement of experts in the field of substance use research (domain experts). We acknowledge a limitation to our approach in that our DAO development team did not include persons with lived experiences of substance use disorders. In the future, it would be important to also involve individuals who use drugs to help develop and refine DAO sections and terms. The DAO can provide a tool and a framework for interdisciplinary collaborative teams to carry this work forward. The DAO ontology has been proven effective in several scenarios, as demonstrated in *Evaluation* section (Section 3). Table 8 summarizes the evolution and improvement of the ontology use according to the needs of the projects. The public health findings described in this document of associated projects, with a focus on person, place, and time, are referenced in Table 8.

**Table 8:** Summary of the drug abuse ontology implemented in projects.

Domain	Related publications	Manuscript section	Data type	Findings reference
Buprenorphine, loperamide, other opioids	Cameron et al [26], Daniulaityte et al [25,82]	PREDOSE^a^ [26]	Web forum data	Figures 4 and 5
User types in marijuana-related posts on social media	Kursuncu et al [28], Lamy et al [31]	eDrugTrends [28-31,96]	Twitter data, web forums, and Bluelight	Table 2
Depression DSM-5	Gaur et al [41]	eDrugTrends [45]	Web forums, Reddit, and Twitter	Tables 3 and 4
Fentanyl, fentanyl analogs, Clustering of dark web vendors	Usha et al [35], Kumar et al [33], Lamy et al [34]	eDarkTrends [46]	Social media and cryptomarket	Tables 5 and 6
COVID-19	Gaur et al [53,88]	COVID-19: public health study [97]	Social media	Figure 10; Table 7

^a^PREDOSE: Prescription Drug Abuse Online Surveillance.

### Principal Findings and Conclusions

In this study, we developed and evaluated the DAO as a framework for identifying concepts, entities, and relationships of interest in social media posts. The DAO developed in this study comprises 315 classes, 31 relationships, and 814 instances with 2 to 4 levels deeper. Our ontology was designed to study social media data, dark web data, and web forums. The DAO is primarily used for knowledge extraction and is broadly applicable to these platforms.

The superclasses of our ontology integrate all concepts regarding health conditions, individual-related, network-related, and society (public policies), sources (dealers, internet, medical, self-produced), spatiotemporal, and substance-related classes. The integrated ontology developed in this study is suitable for analyzing social media posts and dark web posts to understand network-related characteristics, location and time issues, identifying new trends, synonyms, slang items, and new drugs.

Our ontology incorporates terminology not only extracted from DSM-5 but also various terms and slang used on social media and other web posts. The terminology with all the medical terms, synonyms, and slang terms representing all the substances enabled a rich collection of terms in social media and dark web data. Our ontology also helps in topic discovery and entity extraction from social media and dark web data. In addition, we used ontology to extract information in the description of each product in dark web marketplaces to identify substances that are being sold that are not known, such as synthetic drugs, research chemicals, synthetic cannabinoids, and synthetic heroin.

Following well-known software development methodologies (eg, agile methodology), the ontology is constantly being updated according to the needs of current addiction-based research. The DAO stands as a machine-processable resource that describes a collection of addiction domain-related objects and classes, and is growing with the needs of the new ongoing projects. For instance, the current ontology is being enriched with knowledge from the dark web. In future work, the ontology will be linked to other ontologies (eg, MEDDRA [98], a Medical Dictionary for Regulatory Activities) to design the drug abuse knowledge graph. Another research contribution would be to automatically update the DAO with new concepts and properties, inspired by the algorithm that allows users to interactively build topic-specific ontologies using suggestions retrieved from a knowledge graph [99]. Glossary of the terms used in this paper is provided in Multimedia Appendix 1.

## Appendix

Multimedia Appendix 1 Glossary of terms used in this paper.

## Abbreviations

BERT: bidirectional encoder representations from transformers
DAO: drug abuse ontology
DSM-5: Diagnostic and Statistical Manual for Mental Disorders-5th edition
ML: machine learning
NER: named entity recognition
NIDA: National Institute on Drug Abuse
NIH: National Institutes of Health
NLP: natural language processing
ODKG: Opioid Drug Knowledge Graph
PREDOSE: Prescription Drug Abuse Online Surveillance
