# Supplementary material for: Drug Abuse Ontology to Harness Web-Based Data for Substance Use Epidemiology Research: Ontology Development Study
Source: JMIR Public Health Surveill. 2022 Dec 23;8(12):e24938. doi: 10.2196/24938 (PMC9823583; doi:10.2196/24938)
Supplement: Multimedia Appendix 1 [file publichealth_v8i12e24938_app1.docx]

**Multimedia Appendix 1: Glossary of terms used in this manuscript**

**HLF|VLF|FGF**

While training a machine learning model, we organized our feature set into three broad groups: Horizontal Linguistic Features (HLFs), Vertical Linguistic Features (VLFs), and Fine-Grained Features (FGFs). Contextual Features (or embedding of a subreddit post) with Modulations (CFwM) and without Modulations (CFw/oM) are two additional feature set created using Word2Vec on Reddit data. The results regarding this are presented in

**Community Ontology Repository (COR)**

This is the repository of ontologies hosted by ESIP members that would let users try out semantic technologies, understand their benefits, and explore possible applications that used semantic resources. More information can be found at <http://esipfed.github.io/cor/>

**Class|Data Property|Individual count**

These terms are used as the signatures for the imports closure of the active ontology. In other words, the number of distinct classes, object properties, data properties and individuals are mentioned in the ontology. The numbers here include built-in entities, such as owl: Thing if they are explicitly mentioned in the ontology.

**Entity|Concept**

The entity is referred to as an encompassing concept for classes, individuals, and properties. Concept and class are simply synonyms.

**F1 Score**

In the statistical analysis of binary classification, the F-score or F-measure is a measure of a test's accuracy.

**Precision|Recall**

Precision is the fraction of relevant instances among the retrieved instances, while Recall is the fraction of relevant instances that were retrieved.

**DBPedia**

DBpedia is a crowd-sourced community effort to extract structured content from the information created in various Wikimedia projects. For additional documentation refer to https://wiki.dbpedia.org/about

**101 ontology**

The 101 ontology is a guideline to create an ontology and offers step by step process. The tutorial can be found [here.](https://protege.stanford.edu/conference/2004/slides/Ontology101_tutorial.pdf)

**Ontology metrics**

The metrics list the numbers for structures and representation of ontology in Protege as it is the most widely used tool to create ontology. Axioms associate class and properties and are a combination of logical and non-logical attributes. The number of distinct classes, object properties, data properties, and individuals reported are focused on the evaluation of the structure of DAO. More information can be found [here](http://protegeproject.github.io/protege/views/ontology-metrics/) about axioms, concepts, object property, data property, instance etc.

**Protege**

Protege is a free, open-source ontology editor and framework for building intelligent systems

**WebVOWL**

WebVOWL is web application for the interactive visualization of ontologies which is one of ontology visual representations. More information can be found [here](http://vowl.visualdataweb.org/webvowl.html#:~:text=WebVOWL%20is%20a%20web%20application%20for%20the%20interactive%20visualization%20of%20ontologies.&text=The%20VOWL%20visualizations%20are%20automatically,is%20provided%20along%20with%20WebVOWL).

**Owl file**

The W3C Web Ontology Language (OWL) is a Semantic Web language designed to represent rich and complex knowledge about things, groups of things, and relations between things.

**PerfectO methodology**

PerfectO references, classifies and provides tools to encourage Semantic Web Best Practices to achieve Semantic Interoperability by focusing on ontology improvement. The entire documentation can be found at <http://perfectsemanticweb.appspot.com/>.

**[Oops (Ontology Pitfall Scanner)](http://oops.linkeddata.es/response.jsp?uri=http://cor.esipfed.org/ont/~ushanri/DAO) |** [**Vapour**](http://linkeddata.uriburner.com:8000/vapour?uri=http://cor.esipfed.org/ont/~ushanri/DAO) **|**[**Triple Checker**](http://graphite.ecs.soton.ac.uk/checker/?uri=http://cor.esipfed.org/ont/~ushanri/DAO)

These are Semantic Web (SemWeb) validation or documentation tools that help to improve ontologies. Oops detect common pitfalls in ontology automatically and provide recommendations to fix them. The entire documentation of these tools can be found [here](http://perfectsemanticweb.appspot.com/documentation/SemanticWebBestPracticesForDummies.pdf).

**False Positive| True Positive**

A false alarm is also known as a false positive. A false positive is a result that indicates a given condition exists when it does not. For example, cannabis can cause pain when it is not. A true positive is an outcome where the model correctly predicts the positive class. Similarly, a true negative is an outcome where the model correctly predicts the negative class. A false positive is an outcome where the model incorrectly predicts the positive class.

**SEDO**

SEDO stands for Semantic Encoding & Decoding Optimization. It is a procedure to modulate the word embedding (vectors) of a word. SEDO modulates the embeddings of each word in the Reddit content of the user based on the proximity of the word to the DSM-5 category. More information can be found [here](https://dl.acm.org/doi/abs/10.1145/3269206.3271732).

**BRF-CF**

Bootstrap and Bagged Random Forest with Contextual Features. Random Forest is one of the most popular and most powerful machine learning algorithms. It is a type of ensemble machine learning algorithm called Bootstrap or bagging.
